# Development of a database system for mapping insertional mutations onto the mouse genome with large-scale experimental data

**DOI:** 10.1186/1471-2164-10-S3-S7

**Published:** 2009-12-03

**Authors:** Wenwei Yang, Ke Jin, Xing Xie, Dongsheng Li, Jigang Yang, Li Wang, Ning Gu, Yang Zhong, Ling V Sun

**Affiliations:** 1 Institute of Developmental Biology and Molecular Medicine, Fudan University, Shanghai 200433, PR China; 2 School of Life Sciences, Fudan University, Shanghai 200433, PR China; 3 School of Computer Science, Fudan University, Shanghai 200433, PR China; 4 School of Information Technology, Jiangnan University, Wuxi, Jiangsu 214122, PR China; 5 Shanghai Center for Bioinformation Technology, Shanghai 200235, PR China

## Abstract

**Background:**

Insertional mutagenesis is an effective method for functional genomic studies in various organisms. It can rapidly generate easily tractable mutations. A large-scale insertional mutagenesis with the *piggyBac *(PB) transposon is currently performed in mice at the Institute of Developmental Biology and Molecular Medicine (IDM), Fudan University in Shanghai, China. This project is carried out via collaborations among multiple groups overseeing interconnected experimental steps and generates a large volume of experimental data continuously. Therefore, the project calls for an efficient database system for recording, management, statistical analysis, and information exchange.

**Results:**

This paper presents a database application called MP-PBmice (insertional mutation mapping system of PB Mutagenesis Information Center), which is developed to serve the on-going large-scale PB insertional mutagenesis project. A lightweight enterprise-level development framework Struts-Spring-Hibernate is used here to ensure constructive and flexible support to the application. The MP-PBmice database system has three major features: strict access-control, efficient workflow control, and good expandability. It supports the collaboration among different groups that enter data and exchange information on daily basis, and is capable of providing real time progress reports for the whole project. MP-PBmice can be easily adapted for other large-scale insertional mutation mapping projects and the source code of this software is freely available at http://www.idmshanghai.cn/PBmice.

**Conclusion:**

MP-PBmice is a web-based application for large-scale insertional mutation mapping onto the mouse genome, implemented with the widely used framework Struts-Spring-Hibernate. This system is already in use by the on-going genome-wide PB insertional mutation mapping project at IDM, Fudan University.

## Background

Insertional mutations have been playing an important role in biological studies ever since the early development of genetic engineering in the late 1960s (reviewed extensively in [1]). It was first used by bacterial genetics [1]. Later on, its usage was expanded to other organisms by modification of the bacterial system or the discovery of other appropriate natural insertional agents. It has already been successfully applied to the study of gene functions of a number of model organisms such as yeast [2-5], fly [6-10], worm [11-13], plant [14], fish [15-18], and mouse [19-28], and also organisms of economic and/or medical importance such as rice [29], silkworm [30], and mosquito [31]. Insertional mutagenesis has proven itself to be one of the most efficient means for large-scale functional characterization of a number of genomes, including yeast [32], fly [9,33], fish [17], plant [34], and mammal [21,22,28,35-37]. When an appropriate insertion vector is used, a large number of mutations can be produced at low cost and with a high speed, and expression patterns of the inserted endogenous genes are readily revealed. For example, new insertional mutations could be produced from mice carrying transposable elements and are capable of transposition in the germ cells. Progeny harboring individual transposition events can be subsequently mapped and maintained in the absence of the transposase.

More and more large-scale insertional mutagenesis projects are underway to decode genomes of interest. These projects are producing large quantities of experimental data. Simple spreadsheets on personal computers would not meet the demand generated by the current volume of experimental data. Large-scale experimental projects are not handled by isolated individuals/groups, but are instead managed with the same flow production commonly used by car manufacturers. In this method, the whole process is divided into multiple interconnected steps with each step under the charge of a specific research group. This kind of management generates two urgent needs: 1) recording data and exchanging information on a daily basis among the collaborating groups, and 2) extracting statistical information in a timely manner for researchers to grasp the current progress of the project. In other words, an efficient way to record and process data is required. A well-designed and implemented management database is the right choice to meet these needs.

Recently, two databases have been published to facilitate lab management in large-scale mutagenesis projects: the ENU-mutagenesis system for mice [38] and the PACLIMS system used for a rice fungus genome insertional mutagenesis project [39]. Both have been used in their own projects respectively. However, the ENU mutagenesis system does not involve an insertional mutation mapping procedure needed in our project, while the PACLIMS system is only applicable for handling large-scale insertional mutagenesis project carried out in 24- or 96-well plates for cell culture system and microorganisms. In addition, neither satisfies the need of a large-scale project carried out with the flow-control production system.

Currently, a genome-wide *piggyBac *(PB) mutagenesis mapping project managed with the flow control system is underway in the Institute of Developmental Biology and Molecular Medicine (IDM) at Fudan University in Shanghai, China. PB transposon, originally found in the cabbage looper moth *Trichoplusia ni*, is a DNA transposon shown to be an important genetic manipulation tool in multiple organisms including mice [22,40-43]. We have established the PBmice system (PB Mutagenesis Information Center) for archiving, retrieving, and analyzing the resulting data from this project [44], but the published version did not handle either the raw data or experimental flow. This paper presents the MP-PBmice system (insertional mutations mapping system of PBmice), which is developed to meet the demands mentioned above for the efficient management of large-scale projects performed in the flow control fashion: efficient data recording, processing, and exchange. The MP-PBmice system can also be easily adapted to other insertional mutation mapping projects using the flow production mode with large-scale experimental data.

## Methods

### DATA Source

#### Experimental procedure

**Figure 1:**
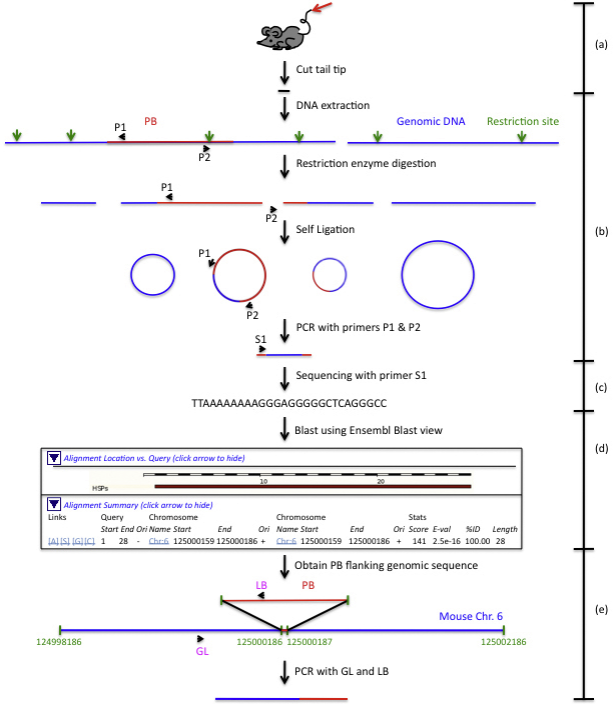
**The experimental flow of mapping insertional mutations in mice with the iPCR method**. (a) Sampling. The tail tips of mice are cut for DNA extraction. (b) iPCR procedure. Extracted mouse genomic DNA will go through restriction digestion, self ligation, PCR with PB specific primers to obtain PB flanking genomic DNA fragment. (c) Sequencing. Obtained PB flanking genomic DNA fragment is sequenced with PB specific primer to gather PB flanking genomic DNA sequence. (d) Mapping insertion onto the genome. The genomic sequence is submitted to Ensembl http://www.ensembl.org/Multi/blastview to blast against mouse genome assembly to obtain location of PB insertion. This part is modified according to the blast result shown in Ensembl blastview website. (e) Genotyping (GT) verification. Primer specific to genomic DNA fragment around PB insertion is designed. This primer is paired with a PB specific primer for amplification of a DNA fragment containing both genomic DNA and PB. Success of this amplification means the insertion is GT verified.

Inverse PCR (iPCR) [45] was chosen in the large-scale PB insertional mutagenesis project. The mapping process of this project runs in the efficient flow-production mode (Figure 1). The whole process is divided into five steps with each step overseen by a designated research group.

#### Mouse tissue and iPCR data

The first step is sampling. In the PB project, mice tissues served as the samples from which the mice genomic DNA was generated. Mice are taken care of by the animal facility group. This group collects the tips of the tails of mice potentially carrying new PB insertions (Inserts) in the genome. Then the iPCR group performs iPCR experiments using these tail tips to obtain amplified fragments carrying the mouse genomic fragment flanking the PB insertion site. These amplified fragments are sequenced subsequently. In this step, the following data are gathered: sample information, experiment status information (fail or successful), DNA electrophoresis gel photos, and the information of the DNA fragment to be sequenced.

#### DNA sequence data

The sequencing of the PB flanking genomic DNA is performed by the sequencing group. For each DNA fragment sequenced, three files are generated to describe the contents and quality of the sequence. These results are passed onto the bioinformatics group to determine the location of the Inserts on the chromosomes.

#### Insertional mutation localization data

The Insert-flanking genomic sequences are obtained from the reliable sequences and submitted to Ensembl and/or UCSC genome browser and/or NCBI for mapping those insertions onto the mouse genome. One example is shown in Figure 1. The locations of the Inserts on the genome and the genomic features of those locations are obtained. For each Insert, a 4 kb sequence with the insertion site in the middle is sent to the Genotyping (GT) group for experimental GT verification.

#### Verification data

The GT group designs GT primers using the above 4 kb sequence as template with the MacVector Software (MacVector Inc.). Suitable primers predicted by the software are submitted to UCSC genome browser to determine its uniqueness in the whole mouse genome. To ensure that there is no accidental sample switch, the animal facility group will collect a piece of the ear for GT verification. The ear tissue comes from the same mouse whose tail tissue was sent for iPCR in the beginning. If the GT verification of one insertion generates a positive result, the mapping process comes to an end for this specific Insert. If the experiment fails, new primers will be designed for a new round of GT verification. Primer information, experiment status information (fail or successful), and DNA electrophoresis gel photos are the three types of data gathered in this step.

### Design and implementation of the MP-PBmice system

The previously published PBmice collect and disseminate the large quantities of the genetic and phenotypic information from this on-going large-scale PB insertional mutagenesis project [44]. It provides a user interface to query and display data related of the published PB insertions in the mouse genome and their characterizations. However, in this published version, neither the raw data nor the experimental flow is touched. In the process of the production of large numbers of insertional mutants and the characterization of their phenotype, large amounts of raw data are produced and recorded daily. There is an urgent need of a mapping system to serve the mutant mapping procedure to ensure detailed and correct data recording and information exchange on a daily basis. We designed and developed the current MP-PBmice system to meet these needs.

#### Database tables

**Figure 2:**
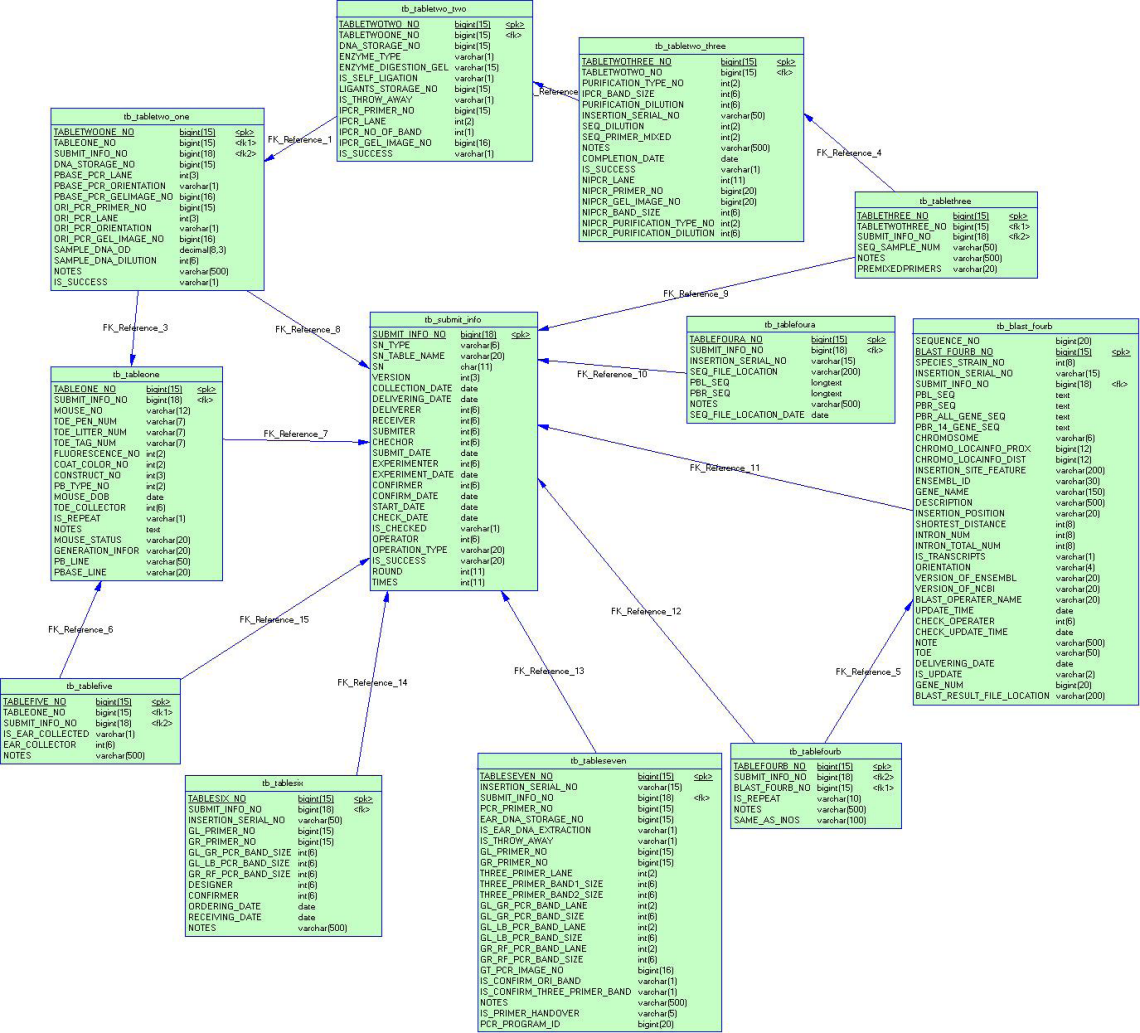
**An example of database tables: Experimental stage tables**.

Experimental data gathered from every step of the project, including future updates, need to be stored in the database. We employ the relational data schema to model these data. Four kinds of tables (one example is shown in Figure 2) are designed in the MP-PBmice to ensure correct daily data recording and exchange.

1. Access-control tables. The function of accessing control in our system is realized through access-control tables including a user table, a role table, a group table, a permission table, and a user-role relation table. The user, role, and group table are used to store the information of every registered user, every defined role, and every designated group respectively. In the permission table we keep the permission status of each data table, such as which table can be read and which table can be updated by a specific role of a specific group. Each user can be allocated multiple roles to carry out corresponding tasks. The user-role relation table archives this relationship between each user and the role(s) allocated to the user.

2. Experimental stage tables. Data created from one step of the whole procedure are often stored in a single table. Data from an earlier stage can be easily accessed in a later stage through foreign keys. For example, if several versions of experimental data of one specific insertion in the same stage are created, foreign keys are able to distinguish the newest version from the old version(s).

3. Constant tables. Certain categories of data are not unique to either each mouse or each Insert generated. For example, the coat color of a mouse falls into a few types: black, white, and others. The experimenters only need to fill in the data via a simple pull-down menu to make the right choice. Constant tables are applied to store these values instead of fixing these options into codes. This designing strategy allows for easy potential updates by updating the data in corresponding constant tables rather than the need to rebuild the whole system.

4. Status table. In the whole experimental flow, mice and Inserts are the key threads. All steps of the experiments are carried out around them and the current status of an Insert is critical to the workflow. The Status table is designed to record the current experimental stage and the status of each Insert in this stage. This strategy smoothens the experimental flow and creates a more efficient query of the stages and statuses.

#### The Model-View-Controller pattern

**Figure 3:**
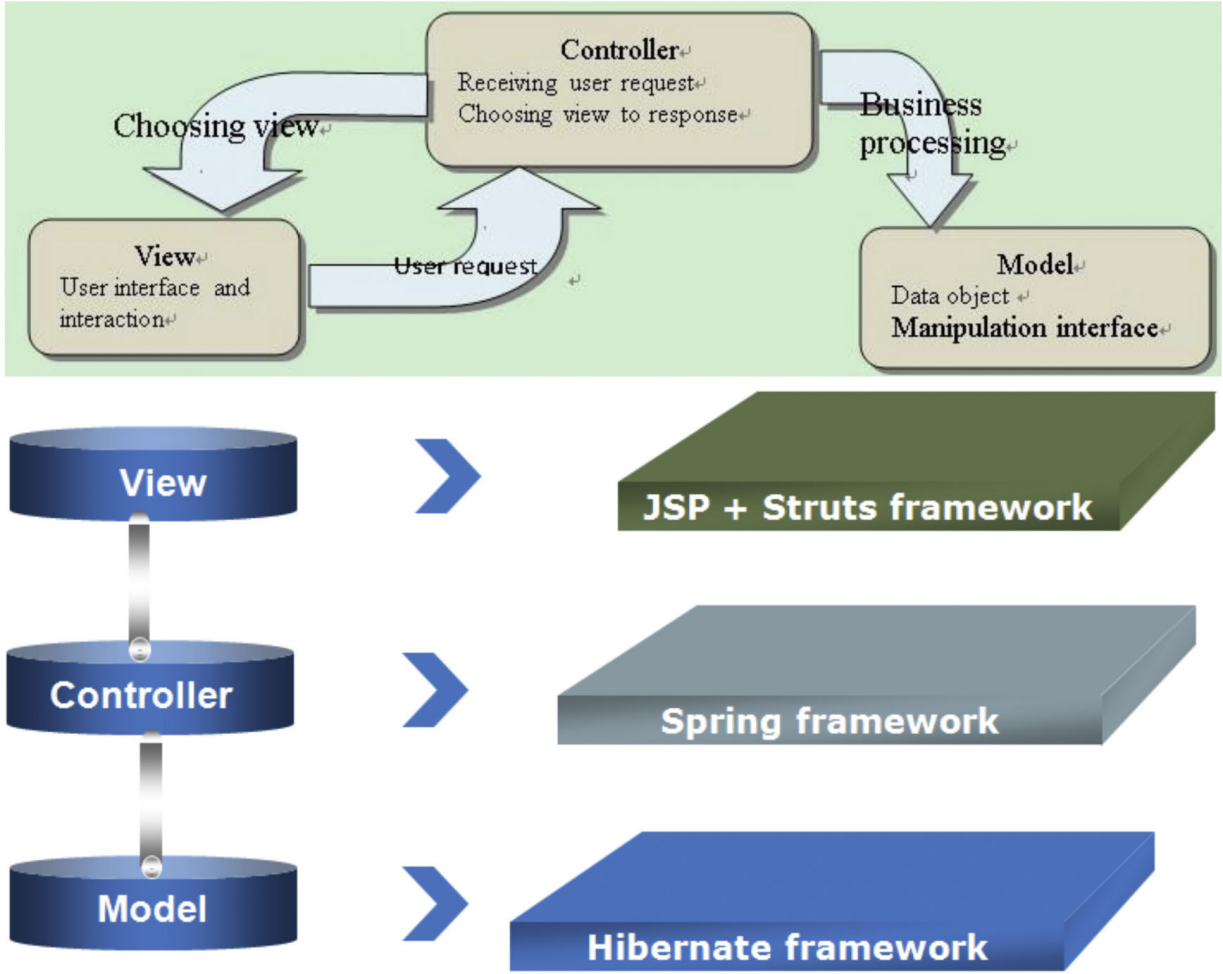
**Relationship between model view and controller**.

The three-layer architectural pattern Model-View-Controller (MVC) http://heim.ifi.uio.no/~trygver/1979/mvc-2/1979-12-MVC.pdf is the choice for the system design in MP-PBmice. The MVC pattern is widely used in software engineering and often used by applications that deal with multiple views of the same data. The basic idea of this mainstream design pattern is to isolate the business logic from user interface considerations. This results in an application that modifies the visual appearance or the underlying business rules in such a way that they will not affect each other (see Figure 3).

In MVC, the Model layer stores the data or information on which the application operates, provides application-specific methods to manage the data, and operates all of the business process of an application. It receives and responds to the requests from the Controller layer and the View layer.

The View layer is a visual representation of the information contained in the Model layer through listening for and reflecting the state updates in the Model layer. A view may change when the information in the Model layer changes, so there can be more than one view for a model.

The Controller layer helps the communication between the Model layer and the View layer by listening to the user inputs and translating these inputs into the data changes in the Model layer. It also instructs the View layer to make changes in the user interface when the Model layer updates.

#### The Struts-Spring-Hibernate framework

We designed and developed the MP-PBmice system with the lightweight enterprise-level development framework Struts-Spring-Hibernate to implement the MVC pattern [46,47]. This development framework is widely used in commerce and would meet the demands from the large volume of experimental data.

In our implementation, the Model layer is also called the Data Access Object (DAO) layer, which is formed by the database models generated by Middlegen http://boss.bekk.no/boss/middlegen/. These database models are composed of Java objects describing the models, operations, and model relationships of the database. DAO performs queries on the database models to obtain the information using Hibernate Query Language supported by Hibernate (v3.0.5) [46]. This framework controls mapping of every schema (table) in the database of MP-PBmice to the underlying relational database MySQL (v5.0.22). Every operation on an object will be automatically persisted into the database. As a transparent back-end layer, it is easy to execute data queries and feature extractions.

The Controller layer is implemented with Spring framework (v1.2.8) [47], a highly customizable and extensible framework that provides basic infrastructures. This framework is also used by DAO to offer the connection pooling and transaction management so that the database connections can be reused by future users. In addition, this framework integrates the service layer containing several services to supply the main functions of the web application developed using Eclipse 3.3.1 http://www.eclipse.org, a freely available Integrated Development Environment (IDE). The View layer of the application is constructed primarily using a combination of Java Server Pages (JSP) (v2.0) and the Struts framework (v1.3.5) [47]. Tiles [48] is also used in the View layer to create reusable view components. Client-side functionality is programmed with JavaScript.

Based on the above architecture, the application was implemented to retrieve, store and operate Mice, Inserts, data-input, and user-management related information as a set of database models defined by Java objects.

## Results and discussion

The MP-PBmice has been developed and is already used for the mapping of the new insertional mutants produced in the large-scale PB project.

### User interfaces

This MP-PBmice system allows users to log in their different roles to carry out corresponding tasks. Here we give one example about how this system works. The complete user menu is available with the source code.

**Figure 4:**
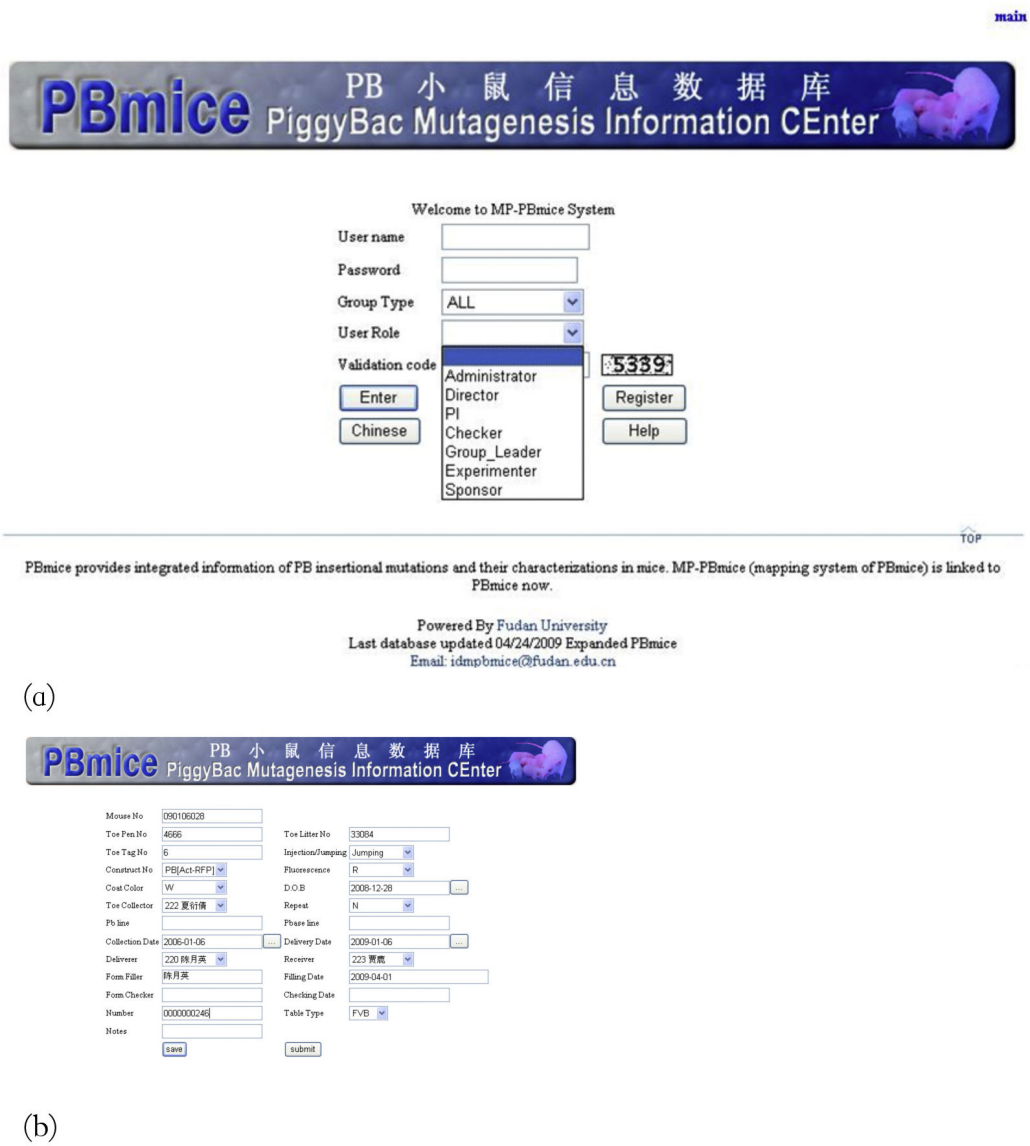
**(a) Login Page**. (b) The Experimental data-filling page.

**Figure 5:**
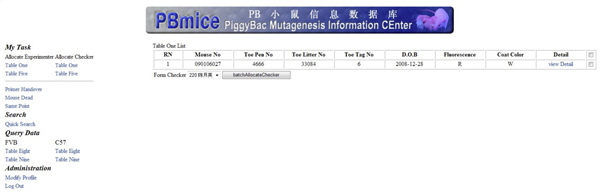
**Checker allocation page**.

**Figure 6:**
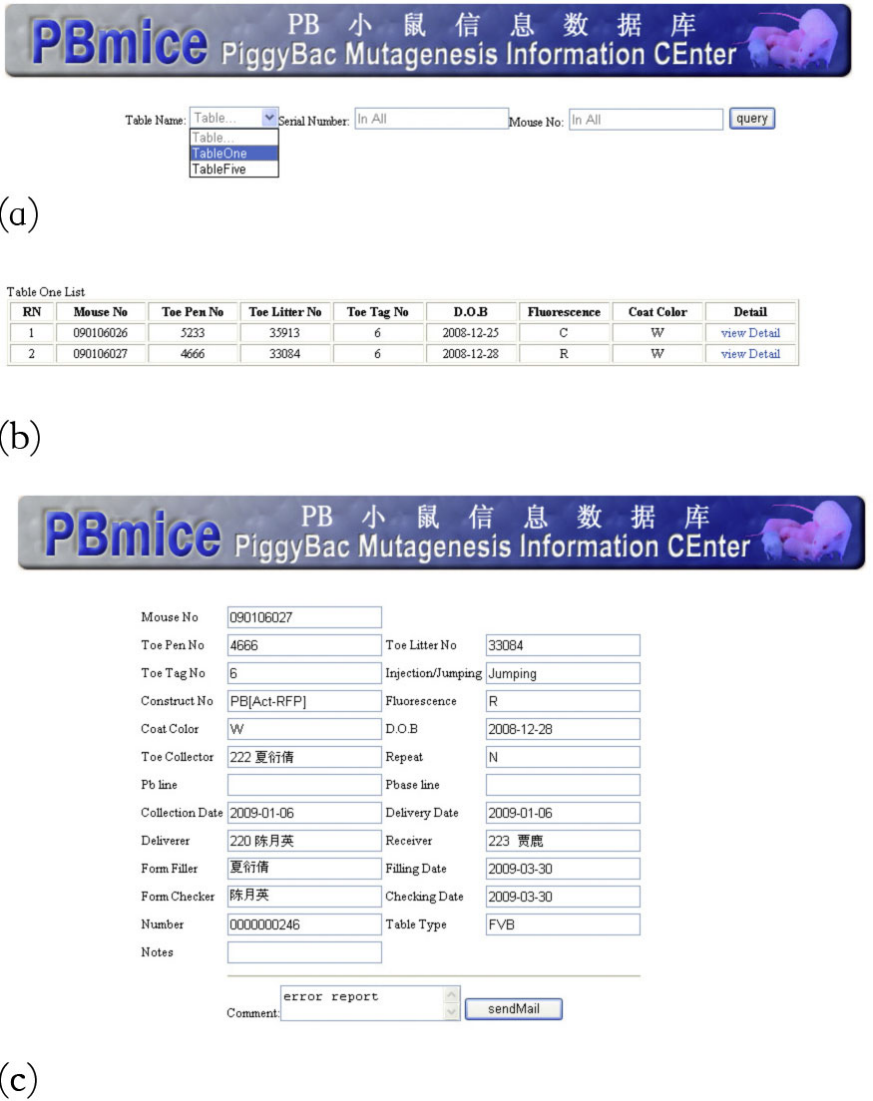
**(a) Quick Search page**. (b) Quick Search result list. (c) Detail information page.

The main entry page is the login page (Figure 4a). A pull-down menu provides the choice of the roles for the specific user. After the Experimenter logs into the system, he/she can fill in his/her own experimental data on the form-filling page (Figure 4b). Once the Experimenter has submitted the data, the Group_leader will allocate a different user as the Checker for this Experimenter to confirm the data (Figure 5). IDM internal users are able to perform quick searches to obtain the detailed information of the experimental data related to his/her work easily (Figure 6a). The search result will be listed and the detailed information will be retrieved through the "viewDetail" linkage (Figure 6b). In the detailed information page (Figure 6c), there is a comment box for remarks and opinions and an email message will be sent to the corresponding PI to report any mistake for correction.

### Main features

#### Strict access control

**Figure 7:**
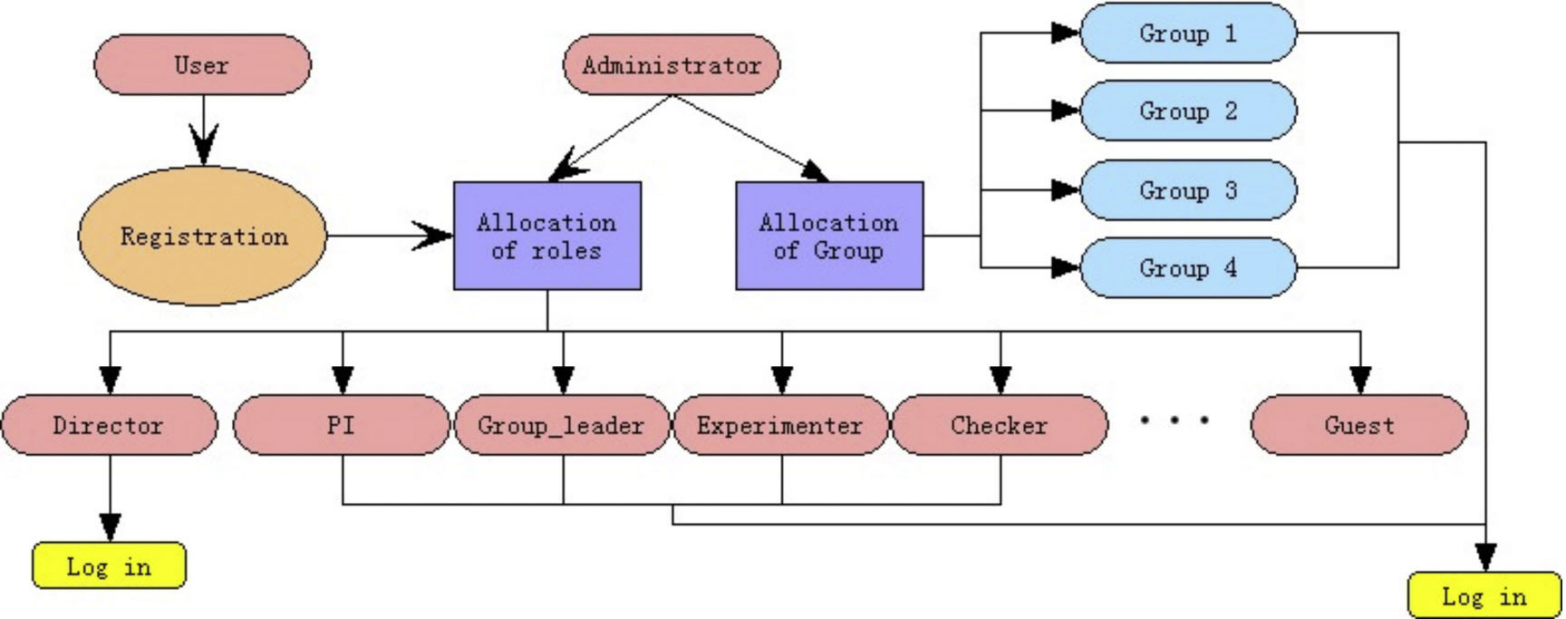
**User registration procedure**.

The MP-PBmice is destined to be a web-based application to meet the needs of concurrent operation of the database by multiple users. An entire strict access control system is introduced into this system to ensure data accuracy. This access control system is divided into three units: 1) user registration, 2) user information management, and 3) user permission management. Each user has only one entry in the user information unit, while each user can be allocated multiple roles in the user permission unit after registration (Figure 7). Different roles in different groups are allocated different permission to perform designated tasks. In each group, the Experimenters perform the allocated experiments, fill in their own data, and submit the data to the database. For each step of the experiment, the Checkers validate the data and are allowed to make modifications only once. If anybody finds an error, she or he may report the mistake through the mistake tracking system. Only the person with the PI role is able to allocate the wrong experiment record to a specific experimenter to correct the data and/or repeat the experiment. The Director role decides to publish the experimental records to the world. All the internet-published data can be accessed without registration. Each operation and the time the operation was performed are recorded into the database. Every error record is also kept in the database as an old version, rather than being replaced. All of these records can be queried by specific roles to show the rate at which error occurred. The strict division of the work and step-by-step checking may reduce the number of mistakes in experimental data submitted, ensure timely data exchange, and improve the efficiency of the whole process.

This control will be developed further to meet more complicated requirements of each role and will generate new roles needed throughout the progress of the project.

#### Workflow control

**Figure 8:**
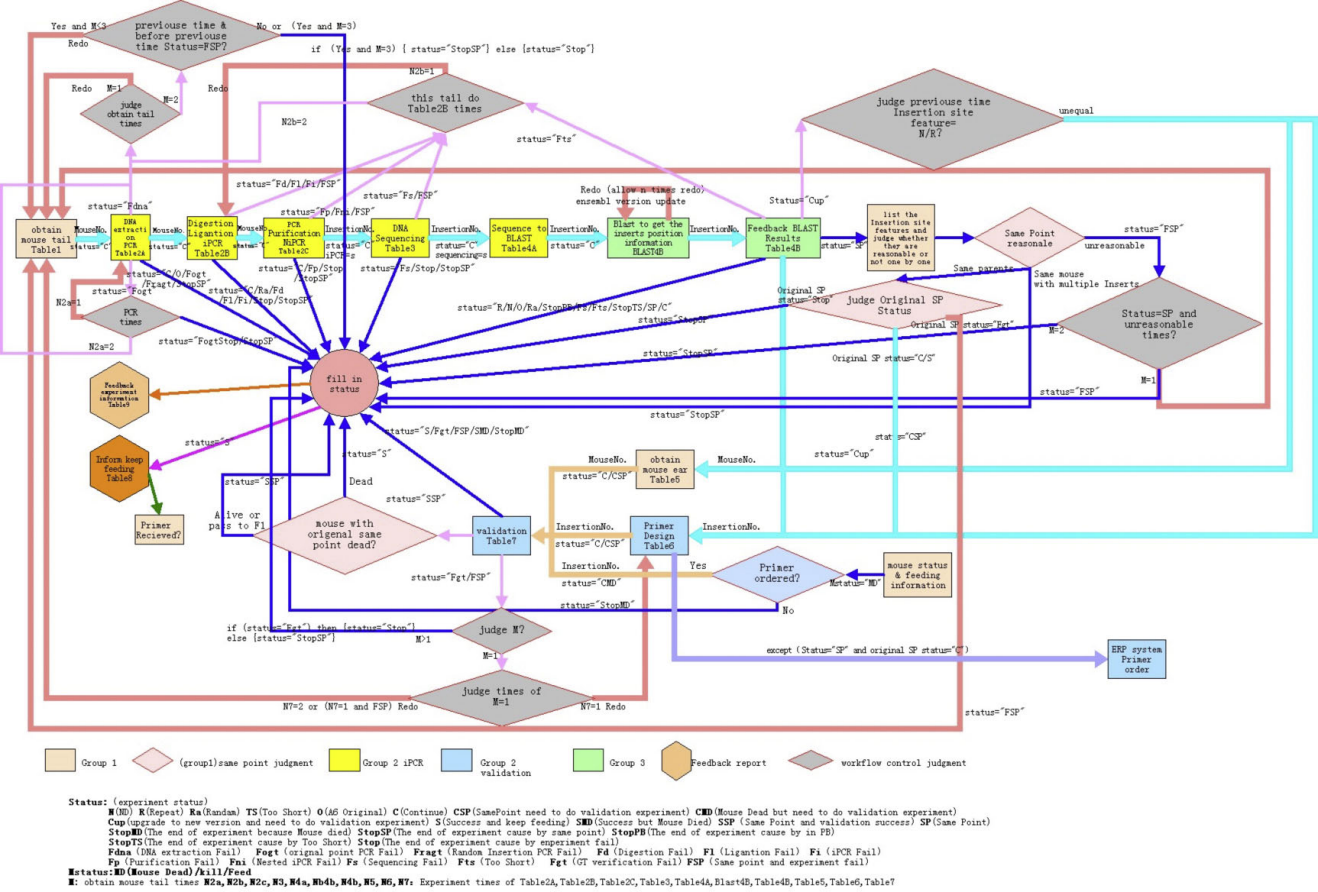
**Workflow of the whole mapping procedure**.

MP-PBmice is developed to have a strict control of the experimental procedure. In practice, each step of the experiment carried out would result in various situations, and the mapping process won't be smooth for every new Insert from the start to the end. Figuring out the complicated branches of the process is the most important part in making the whole project methodically organized. The detailed experimental process and situations that may happen along the way are recorded, discussed, and checked by Directors and PIs of the project. The purpose of this discrete consideration is to work out an approach that avoids the loss of important data or resources as a result of inattentive operation, as well as minimizing the cost of resources and time. A workflow is designed to deal with all kinds of situations that would happen in the experimental process (Figure 8). In each step, an Insert with a specific status defined by the previous experimental result is automatically passed to the corresponding experimental pathway, which notifies the downstream user of his/her coming workloads. Automatic workflow control streamlines the experiments and benefits the process of large quantities of batch analysis. This workflow control also allows a timely report generated by the system for users to visualize the progress of each sample once it is in the procedure. This offers project leaders to analyze the quality and quantity of the experiments so as to improve the protocol in time. With the development of experimental research process, we will improve the system to better support researchers to finish tasks intelligently and efficiently. For example, the system will be able to give suggestions or assessments in certain key functions to researchers through a data analysis system.

#### Expandability

The MP-PBmice system is developed to record all kinds of experimental data generated in a large-scale project of mapping insertional mutations onto the mouse genome. Initially used for mapping onto the mouse genome, it can easily be used for mapping onto any genome, because the only difference will be in the steps of sampling and blasting. For any other genome, the sample will be the corresponding tissue of the organism of interest, and the genome to be blasted is the genome of interest. Either step would generate the same data types as the experimental procedure discussed in this paper. Only the title of sample, location of the Insert (chromosome No. and position) of the table columns in the database need to be changed for other projects. If the thermal asymmetric interlaced PCR (Tail-PCR) [49] or the ligation-mediated PCR [50] is used for mapping the mutants instead of iPCR, only the titles of the table used to record iPCR results need to be changed to the one in use. In both cases mentioned above, the changes are minimal. The MP-PBmice system is easily modified to archive the information generated from other labs for mapping insertional mutations onto a particular genome of interest.

## Conclusion

The large-scale PB insertional mutagenesis project asks for efficient management of the experimental process and data accumulated. The flow production control is more efficient than the mere collaboration among isolated groups, with each group covering the whole process of new mutant production. A database targeting the lab management specifically for large-scale insertional mutagenesis project with flow production control is the key to meeting the demand. In collaboration among three research groups from Fudan University, we constructed the MP-PBmice system to allow researchers to submit data and pass on tasks daily and electronically. The MP-PBmice system was designed and developed with the widely used MPV pattern and lightweight enterprise-level development framework Struts-Spring-Hibernate to ensure the applicability. This system incorporates a strict access-control system and a tight workflow control to ensure the data accuracy and timely information exchange, which are key to the efficiency and success of the project. This system has been in use for five months, and is easily expandable for other large-scale insertional mutation mapping projects.

## Availability and requirements

The software MP-PBmice is freely available at http://www.idmshanghai.cn/PBmice. For other information, please contact the corresponding author.

## Competing interests

The authors declare that they have no competing interests.

## Authors' contributions

WY, JK, and XX designed the architecture of the system. XX, DL, JY and LW designed and implemented the database and all functionalities of the whole system. WY organized the implementation and edited the user guide. YZ, NG, and LS directed the project. YZ conceived the structure of this paper. LS wrote the paper in collaboration with all of the other authors. All authors read and approved the final manuscript.

## Note

Other papers from the meeting have been published as part of *BMC Bioinformatics *Volume 10 Supplement 15, 2009: Eighth International Conference on Bioinformatics (InCoB2009): Bioinformatics, available online at http://www.biomedcentral.com/1471-2105/10?issue=S15.
